# Traumatic Tympanic Membrane Perforation in Children: A Descriptive Cross-sectional Study

**DOI:** 10.31729/jnma.8652

**Published:** 2024-07-31

**Authors:** Apar Pokharel, Chhanya Bhandari, Bibek Sharma

**Affiliations:** 1 Department of ENT and Head and Neck Surgery, College of Medical Sciences, Bharatpur, Chitwan, Nepal

**Keywords:** *pediatrics*, *trauma*, *tympanic membrane perforation*

## Abstract

**Introduction:**

Traumatic perforation of the tympanic membrane in pediatric population is often encountered in primary otolaryngologic clinics or in the emergency department. The objective of the study was to find out the clinical presentation of traumatic tympanic membrane perforation in the pediatric population.

**Methods:**

A cross-sectional study was done from February, 2023 to February, 2024 after obtaining the ethical approval from the Institutional Review Board (2023/114). All the patients aged less than 18 years and presenting with traumatic tympanic membrane perforation were included in the study. Collected data was entered and analysed using Microsoft Excel. Patients were evaluated for demographics, mechanism of trauma and clinical characteristics of ear drum perforation in children.

**Results:**

A total of 384 children aged less than 18 years were included in the study among which 267 (69.53%) were male. Physical assault 109 (28.39%) was the most common cause of tympanic membrane perforation. The most common symptom at the time of presentation was ear block/hearing loss 208 (54.16%). Conductive hearing loss was present in 214 (55.73%) children.

**Conclusions:**

Traumatic perforation of the tympanic membrane resulted mostly from the blunt force trauma especially in male children.

## INTRODUCTION

Children with traumatic ear drum perforations present a multifaceted clinical concern that needs a comprehensive study of prevalence, causative factors, and associated variables. As a result of accidental trauma occurring frequently in pediatrics, the delicate tympanic membrane is at greater risk.^1,2^ A study done in USA reported the prevalence of traumatic tympanic membrane perforation among individuals aged more than 12 years, to be 2.1%.^3^ Another study in Nigeria reported traumatic tympanic membrane perforation in 47.8% among all ear trauma cases.^4^

Despite the potential consequences on auditory function and quality of life, these studies still have little information on clinical characteristics of traumatic ear drum perforations in pediatric population.^3,4^

Therefore, this study aims to find out the clinical presentation of the traumatic tympanic membrane perforation in the pediatric population.

## METHODS

This descriptive cross-sectional study was conducted at the College of Medical Sciences, Chitwan from February, 2023 to February, 2024 after obtaining the ethical approval from the Institutional Review Board (2023/114). All patients less than 18 years, visiting the Ear Nose and Throat Outpatient Department (ENT OPD) or emergency department with traumatic eardrum perforationin the study duration were enrolled in the study. Patients who suffered from tympanic membrane perforation before the traumatic event; past history of ear discharge, ear surgery or congenital ear anomalies were excluded from the study. Informed written consent as well as assent was obtained from all the study participants and their guardians.

Demographic data, mechanism of injury and characteristics of perforation were noted. Symptoms such as earache, tinnitus, vertigo and hearing loss were recorded. The assessment of tympanic membrane was done by endoscope. The size of the perforation at the first visit was graded from 1 to 5, depending on the perceived percentage of tympanic membrane involved: grade 1 = pinhead-sized perforation, grade 2 = less than 25%, grade 3 = 25-49%, grade 4 = 5074% and grade 5 = 75-100%.^4^ The location of the perforation was classified as anterior, posterior or both anterior and posterior based on the imaginary vertical line through the manubrium. All patients underwent audiometric evaluation for assessment of hearing using Interacoustics Diagnostic Audiometer. Pure tone averages were obtained for air and bone conduction thresholds at 250 Hertz (Hz), 500Hz, 1000Hz, 2000Hz, 3000Hz, 4000Hz, 6000Hz and 8000Hz. Hearing loss was calculated and severity of hearing loss was classified as per World Health Organization (WHO).^5^ Pure tone averages were obtained for air and bone conduction thresholds at 250 Hertz (Hz), 500Hz, 1000Hz, 2000Hz, 3000Hz, 4000Hz, 6000Hz and 8000Hz. Frequency of 250Hz, 500Hz and 1000Hz were classified as low frequency, 2000Hz and 3000Hz were classified as middle frequency and 4000Hz, 6000Hz and 8000Hz were classified as high frequency.^6^ A conservative management approach was adopted, but patients with bloody or watery discharge received oral antibiotics to prevent infection. For patients who already presented with middle ear infection, topical antibiotic ear drop was added as well.

## RESULTS

Among 384 children, 267 (69.53%) of them were male The mean age±standard deviation was 9.10±3.97 years. The mean± standard devation duration of presentation was 2.60±1.63 days. The most common symptom during the time of presentation was ear block/hearing loss 208 (54.16%) followed by otalgia 145 (37.76%). On examination of the ear, 185 (48.18%) had perforation in the anterior quadrant and 109 (28.39%) had grade 2 perforation. On hearing assessment, 214 (55.73%) children had conductive hearing loss. Moderately severe hearing loss 69 (17.98%) followed by mild hearing loss 67 (17.45%) were the most common types of severity of hearing loss (Table 1). The hearing assessment of 24 (6.24%) children, aged less than 4 yea years could not be done using pure tone audiogram.

**Table 1:** Clinicodemographic overview of traumatic perforation of tympanic membrane (n=384)

	Variables	n (%)
Gender	Male	267 (69.53)
	Female	117 (30.47)
Site of perforation	Anterior	185 (48.18)
	Posterior	87 (22.65)
	Mixed	112 (29.17)
Types of hearing loss	Conductive	214 (55.73)
	Sensorineural	8 (2.08)
	Mixed	76 (19.80)
	Could not be assessed	24 (6.24)
Size of perforation	Grade 1	77 (20.05)
	Grade 2	109 (28.39)
	Grade 3	84 (21.88)
	Grade 4	61 (15.89)
	Grade 5	53 (13.80)
Complaints of patients	Ear block/hearing loss	208 (54.16)
	Tinnitus	76 (19.80)
	Vertigo	39 (10.15)
	Otalgia	145 (37.76)
	Otorrhea	109 (28.39)
	Could not be assessed	24 (6.24)
Grade of hearing impairment	Normal	62 (16.15)
	Mild hearing loss	67 (17.45)
	Mod. Hearing Loss	62 (16.15)
	Mod. Severe Hearing Loss	69 (17.98)
	Severe Hearing Loss	61 (15.88)
	Profound Hearing Loss	39 (10.16)

Physical assault was the most common cause of traumatic perforation of tympanic membrane accounting for 109 (28.39%) of the total cases with mean age of 12.24 years. Iatrogenic perforation of the tympanic membrane accounted for 62 (16.15%) of total cases (Table 2). Among 109 (28.39%) cases of tympanic membrane perforation due to physical assault, 47 (43.12%) resulted in grade 3 perforation and severe hearing loss was observed in 23 (21.10%) cases. Tympanic membrane perforation due to RTA was seen in 69 (17.97%) cases with mean age of 11.08 years. In RTA cases, grade 5 perforation was observed in 38 (55.07%) cases of which 16 (23.19%) resulted in severe hearing loss. Among 62 (16.15%) cases of iatrogenic tympanic membrane perforation with mean age of 11.08 years, grade 1 perforation was observed in 23 (37.09%) cases. Q-tip injury was seen in 107 (27.87%) cases with mean age of 6.63 years.

**Table 2:** Causes of traumatic perforation of tympanic membrane (n=384)

Cause		n (%)	Gender	n (%)
Blunt Trauma	Physical assault	109 (28.39)	Male	77 (70.64)
			Female	32 (29.36)
	Road traffic accident	69 (17.97)	Male	47 (68.12)
			Female	22 (31.88)
	Water sports	37 (9.64)	Male	22 (59.46)
			Female	15 (40.54)
Q-tip injury	Self-induced	62 (16.15)	Male	46 (74.19)
			Female	16 (25.81)
	Inflicted by others	45 (11.72)	Male	37 (82.22)
			Female	8 (17.78)
Iatrogenic		62 (16.15)	Male	38 (61.29)
			Female	24 (38.71)

## DISCUSSION

This cross-sectional study presented the characteristics of traumatic tympanic membrane perforation in the pediatric population. The 384 children enrolled in the study varied across different age groups aged less than 18 years. The mean age±standard deviation was 9.10±3.97 yearswith a majority being male (69.53%). The duration of presentation was 2.60±1.63 days on average, suggesting that children typically seek medical attention promptly after experiencing symptoms related to ear trauma. The most prevalent symptoms at the time of presentation were ear block/hearing loss 208 (54.16%) and otalgia 145 (37.76%). On pure tone audiogram, conductive hearing loss was the most common findings. The causes of traumatic perforation varied, with physical assault emerging as the most common etiology, accounting for nearly a third of cases. Notably, the etiological profile differed across age groups, with physical assault and road traffic accidents being more prevalent in older children, while Q-tip injury and iatrogenic causes were predominant in younger children.

In alignment with our current study, Rollins et al. conducted a study that also showed a predominance of male (59.1%) in cases of traumatic ear drum perforation.^6^ This demographic trend also is reflected in the findings from other studies where physical assault emerged as the most prevalent cause.^1,7-11^ Furthermore, the findings of our study match with researches done by Lou et al. and Lindeman et al. where in approximately two-thirds of patients sought medical attention within the initial 72-hour window, with 92% and 65% presenting with aural fullness and hearing loss, respectively.^1,12^ These figures indicate the urgency and common symptomatology associated with traumatic ear drum perforations. The anatomical distribution of perforations also is align with our findings. Sagiv et al. identified the anterior quadrant 30 (50%) as the most frequently affected site, mirroring our observations.^7^ However, Orji et al. reported a notable deviation, with 47% of perforations involving both the anterior and posterior quadrants.^13^ Consistent with our investigation, studies done by Orji et al. and Adegbiji et al. showed the predominance of small-sized perforations and a prevalent pattern of conductive hearing loss.^13,14^ Moreover, in study done by Sailesh et al. tinnitus was the foremost presenting symptom in traumatic ear drum perforation cases.^15^

Up to 80% of traumatic perforated tympanic membranes heal spontaneously without the need for any surgical intervention.^16^ However, certain factors can impede this natural healing process. Notably, larger perforations and those located peripherally on the membrane tend to have lower rates of spontaneous recovery.^17^ The edges of these perforations often curl, which can delay healing and may even increase the risk of developing a middle ear cholesteatoma.^17^ Additionally, secondary discharge from the ear frequently fails to close on its own, necessitating further medical attention.^8^ The spontaneous healing of TMs is a complex process influenced by various factors. The size and location of the perforation are critical determinants. Larger perforations create a bigger gap for the tissue to bridge, making spontaneous closure less likely. Peripheral perforations, which are located closer to the edge of the TM, also have reduced healing rates. This is due to the decreased blood supply and mechanical support in these areas compared to the central part of the membrane.^16^ Another challenge in the healing of TMs is the tendency for the edges of the perforation to curl. This curling can interfere with the proper approximation of the edges, thereby delaying the healing process. Furthermore, curled edges can create a niche for the accumulation of keratin and debris, potentially leading to the formation of a cholesteatoma, a destructive growth in the middle ear. The presence of cholesteatoma can complicate the clinical picture, requiring more extensive surgical intervention to prevent damage to the ear structures.^17^ Secondary discharge, often seen with traumatic TM perforations, poses another obstacle to healing. This discharge, which can result from infection or inflammation, hampers the natural closure of the perforation. In many cases, it necessitates medical or surgical treatment to resolve the underlying cause and to promote healing of the membrane.^18^

In essence, the convergence of findings across these studies highlights several key aspects of traumatic ear drum perforations. The male preponderance, in cases with physical assault, prompt medical presentation with characteristic symptoms, and anatomical distribution of perforations collectively provide a comprehensive picture of the condition. Furthermore, the consistent profile of small-sized perforations and conductive hearing loss as common presenting features emphasizes the need for clinicians to be vigilant in recognizing and managing traumatic ear drum injuries. By integrating these collective insights, clinicians can better understand and address the multifaceted clinical manifestations of traumatic ear drum perforations, ultimately improving patient care and outcomes.

This study also had some limitations. As it was a cross-sectional study, follow-up of the patients was not done. As it provided a snapshot of data at a single point of time, causal relationships or temporal sequence of events were difficult to establish. Being a crosssectional study, it also had limited generalizability which meant that the findings might not be applicable to children outside a specific age range or geographical region. There was measurement bias also due to the inability to conduct pure tone audiograms in very young children less than 4 years which could result in underestimating the prevalence or severity of hearing loss in this subgroup.

## CONCLUSIONS

Traumatic perforation of the tympanic membrane resulted mostly from the blunt force trauma especially in male children and commonly presented with symptoms of ear block/hearing loss.
